# Predictors of self-injury cessation and subsequent psychological growth: results of a probability sample survey of students in eight universities and colleges

**DOI:** 10.1186/s13034-015-0048-5

**Published:** 2015-08-03

**Authors:** Janis Whitlock, Kemar Prussien, Celeste Pietrusza

**Affiliations:** Bronfenbrenner Center for Translational Research, Cornell University, Ithaca, NY 14853 USA; Department of Psychology, Cornell University, Ithaca, NY USA; Department of Clinical Psychology, Duquesne University, Pittsburgh, PA USA

**Keywords:** Non-suicidal self-injury, Young adult mental health, Psychological growth, Self-injury cessation

## Abstract

**Background:**

Factors affecting non-suicidal self-injury cessation are poorly understood. The aim of this study was to identify differences between individuals with current and past non-suicidal self-injury (NSSI) in a large probability sample of university students using quantitative and qualitative methods. Predictors of psychological growth related following NSSI cessation were also examined.

**Method:**

The sample included 836 students who participated in a larger online study of well-being at eight U.S. colleges and who reported current or past history of repeated NSSI. The average age of respondents used in analysis was 21.3 years. They were 78.3 % female and 21.7 % male and were 70.7 % Caucasian, 1.4 % African American/Black, 5.5 % Hispanic, 7.8 % Asian/Asian American and 14.7 % other. Analyses tested differences in demographics, NSSI characteristics (e.g. lifetime frequency, number of NSSI functions, NSSI disclosure), formal help-seeking, psychosocial factors, and mental health and trauma histories.

**Results:**

Individuals with current NSSI status were more likely to be female and slightly younger, to report higher NSSI lifetime frequency, more NSSI forms and functions, thinking of themselves as a “self-injurer”, and current psychological distress. Individuals with current NSSI status were less likely to report that self-injury interfered with life, that therapy was useful in stopping, perceiving social support, having a sense of meaning in life, access to more emotion regulation strategies, and life satisfaction. Qualitative data suggested that cessation may be attributable to changes in ability to regulate emotion (62.6 %), self-awareness (38.7 %), and important relationships to others (36.0 %). Psychological growth after stopping NSSI was predicted by more severe NSSI (form and perceived NSSI dependence), having talked about NSSI with others and higher numbers of confidantes, perceived life satisfaction, and a history of suicide action.

**Conclusions:**

These findings add to the still nascent body of literature examining processes related to NSSI cessation. Our results point to the importance of help-seeking and social support, as well as psychosocial processes in stopping NSSI.

## Introduction

### NSSI prevalence, onset, and maintenance

Non-suicidal self-injury (NSSI) is defined as the deliberate, self-inflicted destruction of body tissue without suicidal intent and for purposes not socially sanctioned [1]. NSSI is a common phenomenon, with estimated prevalence rates of 18 % in adolescents [2] to 38 % in young adults [3, 4]. Although most often associated with the term “cutting”, self-injury includes other self-harming behaviors such as intentional carving of the skin, scratching, burning, embedding objects in skin, or swallowing toxic substances. Although the specific behaviors employed as part of NSSI are often confused with suicide, NSSI is, by definition, undertaken without suicidal intent. It does, however, indicate levels of underlying distress that, if left unmitigated, can and sometimes do result in unanticipated severe harm or fatality [5, 6]. Moreover, NSSI is a strong risk factor for concurrent or later suicidal thoughts and behaviors [7–9] and is also often comorbid with a variety of other concerning conditions, such as disordered eating, depression, and anxiety [10–13].

Empirical study of NSSI function generally points to a complex interplay of developmental stage, history of stress or trauma, psychological distress, negative cognition (particularly low cognitive reappraisal, high counterfactual rumination, and low anticipatory rumination), negative effect, and diverting attention away from negative stimulus [14–17]. NSSI is also often reported to increase “good” feelings as well [18, 19]. Recent laboratory-based studies suggest that relief experienced when a painful stimulus is removed, called “pain offset”, may underlie the observed functions [20] and may help explain why it can become habitual. Such findings underscore the complex and dynamic interplay of factors likely to contribute to NSSI maintenance and, most saliently here, cessation.

### NSSI cessation

Although the body of literature contributing to understanding self-injury epidemiology, function, and treatment has grown immensely over the past decade, elucidation of key factors and pathways leading to cessation and recovery is still fairly nascent. Once started, NSSI can last for many years, though it is often cyclical with weeks, months or even years between episodes [21]. The average duration of NSSI among community populations is 2−4 years [22]. Factors associated with NSSI cessation are not well understood. What does exist suggests that demographics, NSSI characteristics, changes in context and/or relationships, reductions in negative effect, and increases in coping capacity may play a role in facilitating cessation [23–26].

For example, in a prospective 1 year study of self-injury, individuals who reported current self-injury reported significantly greater NSSI frequency, more serious wounds, lower cognitive reappraisal, and higher emotional suppression than those who had discontinued the behavior [27]. Similarly, in a study comparing past and current individuals who self-injure, Rotolone and Martin [28] found that compared to individuals who had injured once or more in the past year, those with any self-injury history but who had not self-injured in the past year reported higher family support, self-esteem, resilience, and satisfaction with life. In a similar analysis, Brown and colleagues [23] found few differences in coping style between the past, present and no NSSI history young adults, but did find that individuals with recent self-injury experience reported greater levels of negative emotion than those who had never self-injured. In an examination of factors distinguishing past and current NSSI in high school and college populations, Taliaferro and Muehlenkamp [25] found that depressive symptoms, hopelessness, as well as history of verbal or physical abuse discriminated between the two groups. There were also differences in cessation factors between the two populations studied. For high school students, more anxiety was linked to current self-injury and among college students being non-White, having negative perceptions of one’s weight, a history of dating violence, and/or a same-sex sexual experience were all associated with current self-injury.

In a sample of currently self-injuring community adolescents, Deliberto and Nock [24] examined self-reported reasons for NSSI onset and cessation and found that the most common motivation for wanting to stop was due to perception of NSSI being an unhealthy behavior. Fewer participants reported wanting to stop because of unwanted attention due to NSSI, scarring, shame over the behavior, and because NSSI upsets friends and family. Notably, adolescents who reported that they first encountered the idea to self-injure from a friend were more likely to want to stop for social reasons.

### NSSI growth

Factors which promote cessation of a negative behavior is an important line of inquiry. The concept of *adversity-inspired growth*, however, goes one step further in its postulation of the idea that individuals struggling with mental health challenges or other forms of adversity can, and often do, actively make use of their disorder or challenges to initiate processes of personal transformation and change [29–31]. Research in the area of growth following traumatic events [32] has paved the way for study of the ways in which persistent challenges, such as chronic physical or mental health challenges, facilitate deepening or cultivation of qualities well known to be associated with resilience, hardiness and flourishing [33, 34]. The qualities associated with growth vary some from study to study but tend to include altered capacity to positively reframe events, self-understanding, hopefulness, belongingness, sense of spirituality, appreciation for life, acceptance of one’s life and limitations, quality of relationships and personal strength [35–37].

Studies of growth as a result of NSSI are largely absent but the possibility that struggling with mental health challenges, such as NSSI, may produce a more robust set of experiences than suffering and disability is an area ripe for exploration. In a study of the effects of being asked sensitive questions about self-injurious experience, Whitlock, Pietrusza and Purington [38] found that while 5.2 % of the self-injurious sample found reflecting on these experiences difficult, nearly half (44.9 %) reported benefits to these questions with half of these falling in the “hard but thought provoking” category. In an experimental examination of being asked sensitive questions related to NSSI, Muehlenkamp, Swenson, Batejan and Jarvi [39] found that responding to detailed questions about NSSI did not produce iatrogenic effects immediately or over the follow-up period and may have contributed to positive outcomes.

The mechanisms by which growth takes place are not well understood but tend to assume that encountering chronic adversity tends to challenge and dismantle longstanding psychological patterns or assumptions that are then replaced with new paradigms, perspectives and possibilities [31]. In addition to NSSI severity and therapeutic support linked to NSSI cessation, sharing personal or private thoughts with others may result in positive outcomes when the disclosure is met with empathy and understanding [40]. This may be particularly true when this disclosure leads to clinical treatment, since self-injurious individuals in clinical treatment are less likely to engage in suicidal behaviors, have lower numbers of hospitalizations for suicidal thoughts, and also show lower medical risk in both suicidal acts and self-injurious behaviors compared to those who are not in therapy [41].

### Study aims

The current study aims to address gaps in the cessation and growth literature by comparing differences between individuals with past and current self-injury experience. Through analyses of data drawn from a representative sample of students from 8 diverse colleges and universities, this study is intended to identify factors likely to be salient in NSSI cessation. In light of the existing literature in this area, we anticipate finding differences in the past and present group in a) NSSI characteristics, b) disclosure and formal treatment, c) psychosocial characteristics and d) mental health history. In addition, we extend this analysis and add to the adversity-inspired growth literature by exploring predictors of psychological growth among respondents with past self-injury history.

## Methods

### Sample

The overall sample on which this study is based comes from a study from 8 colleges and universities conducted in the fall of 2006 and early winter of 2007 in the Northeast and Midwest. All but 2 are located in largely urban areas. School size and population varied considerably, ranging from fewer than 2000 undergraduates to over 11,000 undergraduates. The sample was randomly drawn by each university registrar using specialized software. Invitees were sent an e-mail containing descriptive information and a link to the survey. Response rates from each university ranged from 20 to 48 %, with a total of 14,372 respondents (38.9 %). The sample was representative of the overall student populations across all 8 universities in terms of ethnicity, age, and socioeconomic status (SES), although more females than males participated (57.6 % vs 41.7 %). Representativeness was established by comparing study sample demographics (sex, race/ethnicity, and SES) to the student population universe from which the sample was drawn.

For the purposes of these analyses, we restricted our sample to cases for which NSSI was or had been clearly repetitive (>5 lifetime incidents reported) and/ or was limited solely to scab picking or nail biting. After eliminating 12 participants from the full dataset (*n* = 14,372) whose only identified NSSI behavior was scab-picking, a total of 14.0 % (*n* = 2017) of the original sample who had practiced self-injury at least once remained. Twenty five of these reported NSSI but did not respond to self-injury recency (e.g. how long since last injury) items so were not included in these analyses. Of the final eligible sample (*n* = 1992), 42.0 % (*n* = 836) reported having engaged in NSSI on 6 or more occasions and had identifiable past or present NSSI status data; a total of 58.6 % (*n* = 490) had engaged in NSSI in the past year (current repeated NSSI), and 41.4 % (*n* = 346) had not engaged in NSSI in the past year and reported it somewhat or very unlikely that they would injure themselves again (past repeated NSSI).

The average age of respondents used in analysis was 21.3 years. They were 73.8 % female, 26.2 % male and .8 % transgendered and were 71.1 % Caucasian, 2.5 % African American/Black, 4.8 % Hispanic, 8.8 % Asian/Asian American and 12.8 % other. Socio economic status was measured by assessed paternal education: 71.4 % completed college, 13.9 % some college, 11.2 % high school, and 3.5 % less than high school.

### Study design and measures

The *Survey of Student Wellbeing* (SSWB) was administered via a secure Internet server and required 15–30 mins to complete. Response options and, in some cases, order of questions were randomized to avoid selection biases based on response ordering. The study was approved by all participating universities’ Committees for Human Subjects. Multiple response enhancement strategies (e.g., incentives, follow-up reminders, personalized invitations) were employed. Links to local mental health resources were placed on the bottom of every page.

### NSSI characteristics

All NSSI characteristics were assessed using the Non-Suicidal Self-Injury Assessment Tool [19]. An initial screening question for NSSI, “Have you ever done any of the following *with the purpose of intentionally hurting yourself*?” was followed by a list of 19 NSSI behaviors (e.g., “cuts wrists, arms, legs, torso or other areas of the body ” and “carves words or symbols into the skin”) and an “other” option. Participants were then asked questions that assessed NSSI characteristics including but not limited to frequency, function, and age of onset. Lifetime frequency of NSSI (coded as 0, 1, 2**-**5, 6**-**20, >20) was used in these analyses. Individuals who reported using self-injury exclusively as a means of practicing or attempting suicide were classified as not having practiced NSSI. Perceived dependence on NSSI was measured using a 4 item validated subscale included in the NSSI-AT. Two single item measures assessed identification with the behavior (e.g. “I think of myself as a self-injurer”) and perception of NSSI as a problem (e.g. “NSSI is a problem in my life”).

#### Current repeated versus past repeated NSSI status

The primary discriminating variable, current repeated and past repeated self-injury status, was determined by creating two discrete categories of individuals based on NSSI lifetime frequency (only individuals with over 6 lifetime incidents of NSSI were included) and responses to a) length of time since last self-injury and, depending on the response, a follow-up item which asked about likelihood of future self-injury. Individuals who indicated that it had been a year since they last self-injured and that they were unlikely to injure again in the future were coded as “past repeated”; all others were coded as “current repeated”.

#### NSSI disclosure and help-seeking

NSSI disclosure and formal help-seeking measures were also taken from the NSSI-AT. We included an indicator of whether or not the self-injurious respondent has had a conversation with anyone about the self-injury and, for individuals who endorsed this item, the number of such conversations and the number of helpful conversations about NSSI. We also included a set of items related to therapy and formal help-seeking. Five items that were rated on a scale of 1 = agree to 4 = disagree assessing attitudes toward professional help-seeking (e.g. “If I were experiencing an emotional crisis, I could find relief in counseling”.) were summed to form the willingness to seek professional help scale (α = .75) [42]. An indicator was used for whether or not the participant had ever attended therapy for any reason: “Have you ever gone to a therapist (e.g., psychologist, psychiatrist, social worker) to talk about an issue you were having (not including family or couples’ therapy)?” and, if so, and how helpful this had been in stopping NSSI behavior (0 = not at all helpful to 3 = very helpful).

#### Psychosocial measures

Psychosocial measures included a count of the number of people whom the respondent felt he or she could turn to when sad or depressed (0−16), an assessment of the quality of peer social support [43], four items adapted from the McMaster Family Assessment Device reflecting family emotional climate [44], three items linked to the meaning respondents found in life [45], the Limited Access to Emotion Regulation Strategies subscale of the Difficulties in Emotion Regulation Scale [46], and a rating of life satisfaction [47]. All of these were scored using a Likert-type response scale and showed acceptable Cronbach’s alphas in this sample (.73−.92). The number of people to whom respondents turned when distressed was measured by summing endorsed responses to an item which asked, “Who do you feel comfortable getting help from when you feel anxious, sad, or depressed?” Respondents selected all that applied from a list of 23 categories that ranged from friends and parents to therapists and local providers.

#### Mental health and life trauma measures

The mental health and life trauma measures included items intended to measure respondents’ history of trauma and mental health challenges. A count of the number of lifetime traumas (e.g., witnessing or experiencing violence, death of a loved one) was assessed with a modified version of the Life History Calendar [48]. Participants were presented with a list of 12 DSM-IV psychiatric disorders and asked to check which they believed they had suffered from, been diagnosed with, or received medication for. Disorders were summed to create the number of psychiatric conditions suffered measure. The presence of lifetime disordered eating behaviors was assessed with four yes/no items (e.g. “have you ever repeatedly severely restricted your eating?”) [49]. Psychological distress over the last 30 days was measured with a modified version of the K-6 [50]; the “all of the time” response option was omitted. Consequently, continuous K-6 scores ranged from 6–24 and were used rather than categories. Reports of suicidal ideation, behaviors, and attempts were measured using a scale developed by Kessler and colleagues [51], adapted to a web-based format by including an initial screening question, “Have you ever seriously considered or attempted suicide?” Individuals who answered positively were asked to identify specific behaviors engaged in (including ideation), ages, and seriousness of suicide attempts made. Individuals were categorized into three groups based on the most serious level of suicide reported: no suicidal thoughts and behaviors, suicidal thoughts (including ideation, plan, or method), and suicide action (writing a suicide note or attempting suicide). Individuals who indicated that they had considered or attempted suicide but who only then selected that they were not that serious about it were included in the ideation group.

#### Reflections on self-injury cessation and recovery

To further explore the factors that affected self-injury cessation more deeply, we analyzed an additional open-ended question, “If you have stopped altogether (and are confident that you will not intentionally hurt yourself again) please describe why you stopped and what specifically helped you to stop”. This question was only visible to the 346 respondents who were coded as “past NSSI”. Of these, 236 responded to the open-ended item. All of these, 230 were analyzed; six were omitted due to responses considered too cryptic to be coded (e.g. “How do you know it won’t happen again?”).

#### Growth effects of NSSI experience

At the time of the SSWB administration, individuals with NSSI experience who had injured over one year previously and who indicated that they were unlikely to injure again (*n* = 346) were asked to reflect on their self-injury experience by answering the question, “Looking back, how has your experience with intentionally hurting yourself impacted your life, both positively and negatively?” Respondents were offered 12 different dichotomously scored (yes or no) response options that reflected the kinds of responses that individuals interviewed prior to this study had given in response to a similar question. These items empirically factored into three different thematic domains, two of which factored cleanly. The current study uses the Growth scale (e.g. “In thinking/discussing my experience around intentionally hurting myself, I have learned a lot about myself and because of it have mentally/emotionally grown;” “I am now able to help others who intentionally hurt themselves;” “Discussion of my experience around intentionally hurting myself has helped me grow closer to the people I care about”). Factor analyses were performed on the tetrachoric correlation matrix because the indicators are binary and that factor scores were derived using regression. The final reliability coefficient for the Growth scale, using Kuder-Richardson Formula 20 was .66.

### Statistical analysis

All analyses were conducted in SPSS version 22 [52]. Descriptive statistics were run on all study variables by past and current self-injury status (Table 1). Logistic regression with crude odds ratios, and adjusted odds ratios (AORs) with 95 % confidence intervals (CIs) were constructed to examine the multivariate relationships between repeated current and repeated past NSSI status and all independent variables while controlling for demographic variables significant in preliminary analysis: age and sex (Table 1). Linear regressions of growth scores on key study variables were computed for the repeated past NSSI group only (Table 3). To reduce reliance on *p*-values in determining significance [53], we include 95 % confidence intervals along with all effect size coefficients in tables and use all of this information when reporting results and in the discussion section.

**Table 1:** Descriptive statistics and logistic regressions of past repeated NSSI on all study variables

	Current repeated^a^ NSSI		Past^b^ repeated NSSI		Multivariate model^c^
	M (SD)	% (*n*)	M (SD)	% (*n*)	AOR [95 % CI]
Sex^d^					
Male		65.4 (142)		34.6 (75)	1.00
Female		55.9 (340)		44.1 (268)	1.55** [1.11, 2.15]
Age	21.1 (3.4)		21.8 (3.9)		1.05** [1.01–1.1]
NSSI characteristics					
NSSI lifetime frequency					
6–10 times		27.6 (135)		38.7 (134)	1.00
11–20 times		23.3 (114)		27.2 (94)	.79 [.54, 1.15]
21–50 times		22.7 (111)		21.7 (75)	.60* [.41,.90]
> 50 times		26.5 (130)		12.4 (43)	.28*** [.18, .44]
Age of NSSI onset	14.3 (3.5)		14.5 (3.04)		1.01 [.96,1.06]
Number of NSSI functions	6.2 (3.8)		5.08 (3.5)		.92*** [.88, .96]
Number of NSSI forms	3.42 (2.67)		2.96 (2.02)		.93* [ .88, .99]
Perceived dependence on NSSI	2.5 (.99)		2.5 (0.91)		.93 [.60, 1.09]
I think of myself as a self-injurer		30.3 (139)		19.1 (62)	.54*** [.38, .76]
NSSI is a problem in my life	2.38 (1.38)		3.10 (1.48)		1.4*** [1.27, 1.57]
Disclosure & formal help-seeking					
Have had a conversation about NSSI		62.1 (295)		57.3 (189)	0.86 1.01, 1.09]
Number of NSSI conversations^e^	2.3 (1.38)		2.17 (1.17)		0.91 [.78, 1.05]
Number of helpful NSSI conversations^e^	1.63 (0.86)		1.98 (1.10)		1.02 [.95, 1.1]
Willingness to seek professional help	13.09 (3.62)		13.78 (3.99)		1.12 [.98, 1.28]
Ever been in therapy		64.2 (307)		68.9 (230)	1.15 [.85, 1.56]
Helpfulness of therapy in stopping NSSI^f^	1.64 (0.86)		1.97 (1.11)		1.45*** [1.19, 1.76]
Psychosocial factors					
Number of people can turn to when distressed	3.20 (2.37)		3.43 (2.29)		1.03 [.97, 1.10]
Quality of social support	10.42 (2.02)		10.86 (1.77)		1.25*** [1.10, 1.41]
Family emotional climate	12.24 (4.71)		12.17 (4.74)		0.99 [.97, 1.02]
Found meaning in life	20.84 (5.57)		21.95 (6.38)		1.21** [1.06, 1.37]
Emotion regulation strategies	26.31 (7.43)		29.48 (6.88)		1.39*** [1.23, 1.58]
Life satisfaction	20.02 (7.39)		21.91 (7.37)		1.22** [1.07, 1.40]
Mental health and life trauma					
Count of traumatic life events	2.30 (1.73)		2.24 (1.67)		0.96 [.88, 1.05]
Perceived suffered psychiatric condition		62.0 (304)		69.1 (239)	1.29 [.95, 1.73]
Disordered eating behaviors		49.8 (244)		49.7 (172)	.96 [.72, 1.27]
K-6 score	15.4 (3.99)		13.6 (3.67)		.88*** [.85, .92]
Suicidal thoughts or behaviors					
No suicidal thoughts or behaviors		45.4 (221)		40.8 (141)	
Suicidal thoughts only		36.4 (177)		41.7 (144)	1.25 [.92, 1.7]
Suicide behaviors		18.1 (88)		17.3 (60)	1.1 [.74, 1.65]

*NSSI* non-suicidal self-injury, *CI* confidence interval, *AOR* adjusted odds ratio

^*^ *p* < .05, ^**^ *p* < .01, ^***^ *p* < .001

^a^repeated indicates 6 or more lifetime NSSI episodes

^b^Past specifies individuals who have not practiced NSSI for at least one year and indicate that they unlikely to injure again

^c^Derived from one logistic regression model per row that controls for sex and age

^d^Frequencies do not sum to 100 % due to missing data

^e^of participants who had ever had a conversation about NSSI

^f^of participants who had ever been to therapy

Qualitative data were analyzed using the constant comparative method [54] to identify salient themes and was analyzed in two waves, once to derive overarching conceptual categories and related subthemes, then to apply derived codes. The first step was conducted collaboratively and iteratively with input from all authors and the second step, application of codes to data, was conducted by two independent coders familiar with the data. Responses to the open-ended item were then analyzed by two independent coders who systematically reviewed responses and, once the initial set of observations had been reviewed, key emergent themes discussed, and coding scheme determined, thematically grouped clusters were identified and were give a primary and, if warranted secondary code. Coders agreed on all but 15 % of the primary categories and subtheme assignments. Disagreements were resolved by discussion and consensus.

## Results

Preliminary bivariate analysis of difference between past and current repeated self-injury participants by demographic characteristics revealed no differences except that those in the past repeated NSSI group were more likely to be female than male and were slightly but significantly older (*M* = 21.83 years, *SD* = 3.96) than the current repeated NSSI group (*M* = 21.16 years, *SD* = 3.46), *F*(1828) = 6.34, *p* = .012. There were no statistically significant differences in NSSI group by race/ethnicity, father’s education level (used as a proxy for socioeconomic status), and sexual orientation.

Descriptive statistics for all study variables by NSSI past and current repeated groups along with multivariate analyses controlling for sex and NSSI frequency are shown in Table 1. Of NSSI characteristics, lifetime NSSI frequency of 21–50 and more than 50 times, the number of NSSI functions, identification as someone who self-injures, and believing that NSSI is a problem predicted differences between in current and past NSSI group status. Specifically, individuals with higher NSSI lifetime frequencies were less likely to have stopped. This association appeared to be dose-dependent, with 50 or more lifetime NSSI episodes showing lower odds of stopping NSSI than lifetime frequency of 21–50. Examination of effect sizes and confidence intervals also showed that past NSSI status was predicted by fewer number of NSSI functions endorsed (AOR .92, 95 % CI, .88–.96), fewer number of NSSI forms used (AOR .93, 95 % CI, .88, .99), less likelihood of thinking of oneself as a self-injurer (AOR .54, 95 % CI, .38–.76), and of greater acknowledgement of perceiving NSSI as a problem in one’s life (AOR 1.40 95 % CI, 1.27–1.57). Individuals who had ceased self-injuring were also more likely than the current self-injury group to be female (AOR 1.55, 95 % CI, 1.11, 2.15) and to be slightly older than the current self-injury group (21.8 versus 21.1 years). There were no differences in age of onset or perceived dependence on NSSI between past and current NSSI groups.

Current and past NSSI status was not predicted by any of the disclosure measures, except that individuals who had stopped were more likely than their currently injuring peers to report that formal therapy was helpful in cessation (AOR 1.45, 95 % CI, 1.19–1.76). The psychosocial measures were consistent predictors of NSSI cessation. Specifically, individuals who had stopped reported higher quality social support from peers (AOR 1.25, 95 % CI, 1.10–1.41), more found meaning in life (AOR 1.21, 95 % CI, 1.06–1.37), greater life satisfaction (AOR 1.22, 95 % CI, 1.07–1.40), and more effective emotion regulation strategies (AOR 1.39, 95 % CI, 1.23–1.58). The only mental health history measure that was useful in discriminating between the two groups was current psychological distress (K-6), where current NSSI status was associated with greater current (last 30 days) psychological distress.

The next analysis used comments made by individuals who have stopped self-injuring to understand factors ascribed to their successful cessation. Results of these analyses are shown in Table 2.

**Table 2:** Why stop? Attribution categories, sub-themes, and examples

Category/subtheme (% of all respondents with this as a primary or secondary code)	Example
Connection with others (36 %)	
Positive connections 23.9 %	“I entered into a loving relationship”
	“Some of my high school friends were really concerned about what they knew, and talking to them helped a lot”
Negative effect on cared for others 5.2 %	“I stopped because of the people that loved me at the time. I wasn’t just hurting myself, but I was hurting the people that cared about me. That was hard for me to understand, but once it clicked I was done.”
Removal of negative relationships 6.9 %	“Space away from family/frustration.”
	“I moved away from the cause – my parents.”
Professional/Therapeutic Support (7.4 %)	“Through the program of recovery that I follow for my substance abuse problem (AA) and through the assistance of my therapist/psychiatrist, I have learned that I am not alone in those feelings and have been shown real solutions for the uncomfortable feelings I have.”
Emotion Regulation (62.6 %)	
Self-awareness 38.7 %	“I also developed more of a sense of proportion: by which I mean, firstly, that I started to realize that however bad I feel, it’s probable that I’ll feel better at some point in future, and that I should the not act in ways that might permanently diminish my happiness; and secondly, that my emotional distress is minor in comparison to that of many other people.”
	“I gained self-esteem and wasn’t so hard on myself anymore”
Coping skills (tools/behaviors or direct differences) 23.9 %	“I realized I could cope with my emotions in less destructive ways.”
	“I practice martial arts and work out to focus my mind, being able to spar with someone else helps too.”
Life circumstances changed (10.7 %)	“I am happy with my life now, there is no reason for me to be nervous or scared or angry all the time”
Fear of consequences (14.2 %)	
Environmental/Social 3.5 %	“The school made an official policy against the scars and penalized students for doing so. This is when I stopped doing it.”
Physical effects 10.7 %	“I cut too deeply and scared myself.”
	“I don’t want to have scars; they’re ugly.”
Maturity (26.9 %)	“I grew out of it and realized I didn’t need attention that badly.”
	“Most of it I attribute to maturing, to growing out of the raging hormones of adolescence.”
Minimal life effects (15.7 %)	“It doesn’t really matter to me that much whether I do it again or not. Now I don’t ever feel the need to, but I wasn’t addicted and I had no serious incidents.”

As a whole, respondents identified increases in emotional regulation skills as the primary driver of NSSI cessation, with 62.6 % of all respondents receiving at least one *emotion regulation* code. Many also reported growth of self-awareness, with 38.7 % of respondents receiving at least one of these codes, and 23.9 % identified changes in coping skills or tools. The next largest category was in the area of relationships, with 36 % of all respondents receiving at least one *connections with others* code, with 23.9 % indicating that caring friends or loved ones were a strong factor in the decision to stop. *Maturity* was the third dominant theme category with 26.9 % of all respondents identifying that they simply “grew out of it” in some way. Notably, despite the fact that the respondents included in these analyses had all repeatedly self-injured, 15.7 % indicated that the practice had minimal impact on their lives. Only 7.4 % identified therapy as a clear factor in their cessation.

### Growth orientation in NSSI cessation

The second model was designed to identify the factors among those used in the cessation analysis that predicted a growth orientation as a result of NSSI experience. This analysis was restricted to the 230 participants who met criteria for past repeated NSSI and who answered this question. A simple count of endorsed items showed that about 67 % reported endorsing no growth items, 20 % reporting at least one growth item, 8 % reporting two growth items, and 5 % reporting all three NSSI growth scale items.

The bivariate and final multivariate model controlling for all items significant in the bivariate model is reported in Table 3. Bivariate analysis suggested that, as a group, NSSI characteristics, disclosure and help seeking and psychosocial factors were most useful in predicting growth scores. Examination of effect sizes and confidence intervals showed notable effects for multiple secondary NSSI characteristics: number of lifetime incidents, number of NSSI forms and functions and perceived dependence on NSSI. Also notable were whether one has had a conversation about NSSI with someone, number of individuals one perceives e/she can turn to when anxious, sad or depressed, current life satisfaction, and having a history of suicide-related behavior.

**Table 3:** Ordinary least squares regression of growth measure on disclosure, formal help-seeking and psychosocial measures

	Bivariate model ^a^		Multivariate model ^b^	
	Unstd. b [95 % CI]	Std b	Unstd. b [95 % CI]	Std b
Sex, female	0.36^**^ [.12, .61]	.16	.13 [-.17, .42]	.05
NSSI characteristics				
NSSI lifetime frequency	.13^***^ [.03, .22]	.14	-.03 [-.15, .09]	-.03
Age of NSSI onset	.03 [.01, .06]	.09	--	--
Number of NSSI functions	.07^***^ [.04, .10]	.27	.01 [-.03, .05]	.04
Number of NSSI forms	.10^***^ [.05, .15]	.21	.08^*^ [.02, .14]	.20
Perceived dependence on NSSI	.32^***^ [.20, .43]	.31	.21^**^ [.07,.34]	.20
Disclosure & formal help-seeking				
Have had conversation about NSSI	.52^***^[.32, .72]	.27	.25^*^ [.007, .48]	.12
Willingness to seek professional help	.08 [.00, .17]	.10	--	--
Ever been in therapy	.18 [-.04, .40]	.10	--	--
Perceived helpfulness of therapy in stopping NSSI ^c^	.12 [.00, .25]	.14	--	--
Psychosocial factors				
Number of people can turn to when distressed	.10^***^[.06, .14]	.24	.07^*^ [.01, .13]	.12
Quality of social support	.14^**^ [.05, .24]	.17	-.004[-.08, .15]	-.004
Found meaning in life	.17^**^ [.05, .23]	.17	.03 [-.08, .15]	.04
Life satisfaction	.20^***^ [.11, .29]	.23	.15^*^ [-.02, .28]	.16
Emotion regulation strategies	.9 [-.02, .17]	.09	--	--
Mental health history				
Count of traumatic events	.05 [-.02, .11]	.14	--	--
Number of perceived psychiatric conditions	.14 [-.09, .36]	.07	--	--
Disordered eating behaviors	.07 [-.14, .27]	.04	--	--
Psychological distress in past 30 days (K-6)	-.02 [-.05, .01]	-.08	--	--
Suicide thoughts and behaviors	-	-	--	--
Suicidal thoughts	.12^*^ [-.09, .34]	.06	.17 [-.9, .43]	.09
Suicide behaviors	.50^***^ [.23, .77]	.20	.37^*^[.07, .68]	.15

*NSSI* non-suicidal self-injury

^*^ *p* < .05, ^**^ *p* < .01, ^***^ *p* < .001

^a^Derived from one ordinary least squares (OLS) regression model for each predictor

^b^Derived from one OLS regression model with all significant bivariate predictors included

^c^Of participants who had ever been to therapy

When all independent variables significant in the bivariate model are entered in the multivariate model, six show robust effect sizes when all parameters of interest are considered: perceived dependence on self-injury (*unstd β =* .22, 95 % CI = .11, .34, *p* <. 001), having had a conversation with someone about NSSI (*unstd β =* .29, 95 % CI = .06, .51, *p* <. 01), number of self-injury forms (*unstd β =* .06, 95 % CI = .01, .12, *p* <. 001), number of individuals one confides in (*unstd β =* .06, 95 % CI = .01, .11, *p* <. 01), perceived life satisfaction (*unstd β =* .15, 95 % CI = .04, .25, *p* <. 01), and history of suicide-related action (*unstd β =* .34, 95 % CI, =.06, .63, *p* < .01). As a whole, the multivariate model explained a significant amount of the proportion of variance in growth scores, *R*^*2*^_*adj.*_ = .21, *F* = 13.01, *p* < .001.

### Comment

Understanding factors associated with NSSI cessation is a nascent but important empirical endeavor. Consistent with the small but growing body of research in this area, largely conducted with college populations [23], we find that stopping NSSI behavior is associated with a variety of factors across several domains. In general, cessation is related to sex (notably, current self-injury reporters are more likely to be male than female) and, to a lesser degree, current age (past self-injury reporters are slightly older; this is probably not surprising in light of the fact that they would have had, overall, more time to stop. It is important to note, as well, that both age and number of forms show confidence intervals that suggest less than robust effects), the intensity of the NSSI, the perceived value of therapeutic and presence of social support, psychosocial characteristics and current psychological distress. The pattern of findings in the cessation analysis related to primary NSSI characteristics suggests that more entrenched self-injury practices (as measured by primary NSSI characteristics) are a key factor. Greater number of NSSI incidents, forms used, functions reported and identification as a “self-injurer” are all associated with current NSSI while greater acknowledgement of self-injury interference with life is associated with past self-injury. The role of age with regard to cessation is interesting. Although it makes sense that individuals in the cessation group would be older because they had had more time to stop, age of onset was not a factor and the statistical effects for age in the reported models is less than robust. In preliminary models, not all reported here, we also examined length of time self injuring and found that it did not contribute to cessation either. Psychosocial factors also clearly emerge as important. Feeling connected to others, possessing a broader array of emotion regulation techniques, and reporting a sense of meaning and satisfaction in life all enhance the likelihood of stopping. In terms of effect size, reporting current global psychological moderate or elevated distress is a strong predictor of current NSSI.

Qualitative exploration of a question designed to assess how individuals with past NSSI experience understood why they stopped generally reinforce the quantitative findings. Interestingly, participants talked largely about what they perceived changed in their lives over time to support cessation. In line with the quantitative analyses, they identified a) acquisition of emotion regulation strategies (62.6 %), b) positive connections with others (36 %), c) general “maturity” (26.9 %), d) fear of consequences (14.2 %), e) general changes in life circumstances (10.7 %), and f) professional therapeutic support (7.4 %). Just over 15 % responded that stopping was easy because it was not a big part of their lives to begin with. We find it notable that, as with the quantitative findings, formal therapy was a factor but not a leading identified element of cessation; more salient seem to be enhanced self-awareness and emotion regulation skill acquisition coupled with changes in contextual factors.

Although research on self-injury cessation is scarce, our findings are consistent with other studies of cessation. For example, in a multi-wave longitudinal study of self-harm over time, Moran and colleagues [55] found that natural developmental processes (what is referred to as “maturity” here) play an important role in the cessation process. Similarly, other studies have identified the role of NSSI severity as a factor contributing negatively to cessation (e.g. more frequent and physically deleterious NSSI; [27]). The current study reinforces the role of NSSI severity and also suggests that number of NSSI forms and functions also play an important role. This and other studies [25] also find that higher psychological distress is also an impediment to NSSI cessation.

The role of psychosocial variables is more nuanced. Taken as a whole, these findings suggest that individuals who successfully cease NSSI behavior may do so because they develop higher-order reflective cognitive and emotional capacities. In their investigation of the role of emotion and coping in NSSI cessation, Brown and colleagues [23] did not find significant differences in coping skills, per se, between past and presently self-injuring participants but did find differences in perceived levels of negative emotion. Rotolone and Martin [28] documented differences in perceived family support, self-esteem, resilience, and satisfaction with life. Tatnelll et al. [26] found that a combination of intrapersonal and interpersonal factors contributed to cessation, with capacity for cognitive reappraisal playing a significant role. In the current study, both emotions and emotion-linked perceptions (cognitions) were important. For example, cessation was not predicted by engagement in therapy, but generally being open to therapy and, more specifically, viewing one’s personal therapy positively. Similarly, self-injurious individuals who had stopped were also more likely to perceive NSSI as a problem in their lives, and to have found a sense of meaning and life satisfaction. They were also likely to report more diverse strategies for managing difficult emotions than their currently self-injurious peers. Interestingly, although those who had stopped identified emotional regulation as a key area of change in the qualitative data, they were more likely to talk about enhanced self-awareness rather than the adopting of new coping skills in particular. Notably, over a quarter of respondents in the current study identified natural processes associated with maturity in cessation but age of onset did not contribute to explaining the difference between the current and cessation NSSI group. This suggests that drivers of change may be closely linked to the development of new cognitions, emotion and emotional regulation processes in ways that are not linked exclusively to age.

Extant literature also identifies social/contextual factors as important for NSSI cessation. In a study of adolescent advice for teen NSSI cessation, Berger, Hasking, and Martin [56] found that having non-judgmental parents and teachers to talk to was related to improvements in parent-child relationships, referrals to professionals, and reduced school pressures. Tatnelll et al. [26] found family support to a critical factor in cessation. Findings from the current study, however, suggest that enhanced emotional and social awareness and skill and an increased willingness to make use of social supports such as therapy and loved ones, may also be relevant to NSSI cessation. For example, while our respondents qualitatively identified connections with others as the single most powerful contributor to cessation, the quantitative results suggest that it is not the mere availability of others or supportive contexts that matter, but rather the ability to positively perceive and make use of these connections that matters most. It is worth noting that rates of NSSI disclosure are quite variable. Between 31 % and 89 % of adolescent NSSI samples report disclosing their self-injurious behavior to someone [3, 57] and this is most often peers [58–60]. Despite the reliance on peers, respondents tend to rate conversations with friends as less helpful than conversations with parents or other adults [61] suggesting that while confiding in someone is important, confiding in an adult capable may be most important.

The current study also was designed to extend our understanding of NSSI cessation beyond the process of stopping and into the after effects of repeated self-injurious experiences. Toward this end, we examined respondent scores on a measure of psychological growth as a result of self-injury. This scale was intended to measure the perceived effects of NSSI experience, following cessation, along a dimension of perceived growth as a person and utility in helping others. Findings from this aspect of the study suggested that approximately one-third (33 %) of the past self-injury sample perceived any benefit to the experience with 5 % indicating growth in all areas measured. Examination of the factors that explained variation in growth in the final multivariate included six key factors: number of NSSI forms, degree of perceived dependence on NSSI, conversations with others about NSSI experience, number of confidantes one can turn to when distressed, experience with suicide-behavior (beyond suicidal ideation), and sense of current life satisfaction. Of note, conversations with others about NSSI experience, having felt a high dependence on NSSI, and experience with suicide-behavior were the most powerful predictors of growth which suggests that there may be something in the very intensity of adversity coupled with the benefits of processing difficult experiences with others that contributes to a growth orientation. Isolating other important contributors, such as personality and temperament factors, optimism/pessimism and/or fixed versus flexible cognitive orientation would be a welcome extension to this line of inquiry and may contain useful implications for intervention and treatment.

### Implications

The current analyses are unique in their objective and approach and contribute to the fledgling body of knowledge describing the particularities of NSSI cessation. They are also unique in their contribution to understanding factors that facilitate a growth orientation among those with a history of NSSI. They are not, however, without limitations. While we were able to capitalize on the sample size and power for analyses, our ability to accurately account for temporal factors in the processes of interest was limited. As such, although the sample size permits more comprehensive analyses, the comparisons in these data are drawn from one time point and based on retrospective data (current versus past NSSI history). Similarly, self-injury is very cyclical and people may stop for long periods of time and then start again. Our decision to designate the cessation group as individuals who had stopped for a year or more and who reported themselves not very likely to self injure again may more accurately reflect cessation intention then full recovery. Future studies may include using a longer cessation, such as three years, as a more absolute marker of recovery. Lastly, the current study was primarily conducted in a college population and may not be generalizable to other populations.

The fact that the number of NSSI functions endorsed and current psychological distress differentiated past and present repeated self-injury suggests that the magnitude of reliance on the behavior may supersede the particular function in predicting cessation capacity. Potentially mutable indicators are those related to enhanced self-awareness: acknowledging that NSSI is a problem and NSSI as a factor contributing to a sense of meaning and satisfaction in life. Interestingly, satisfaction in life is also a factor in predicting psychological growth as is reporting higher numbers of social confidants and support. The fact that psychological growth was also predicted by greater levels of NSSI dependence while injuring as well as by a history of suicide behaviors also lends credibility to the idea that enhanced self-awareness and social support may be primary factors in the recovery and growth process.

Making and maintaining connections to others, through a willingness to seek and actively use therapy (in the case of cessation) as well as an openness to talking to others about NSSI and confiding in multiple others when distressed, is another clearly important factor in the cessation and growth process. What predicts help-seeking, however, is less clear. Help-seeking is positively associated with the frequency of NSSI [59]; however, adolescents and young adults with only one occurrence of NSSI are more likely to engage in help-seeking than those with recurrent incidents [62, 63]. Secondary characteristics, such as individual perception that NSSI is a problem, also play a role. Fortune, Sinclair, and Hawton [64] found, for example, that perception of the behavior in the moment (i.e., premeditated or spur of the moment), motivation to act, perception that something can and should be done, and desire for help all differentiated help-seeking from non-help seeking. Other factors that contribute to help-seeking include knowledge of NSSI as a phenomenon, becoming consciousness of being in need of help, and the support of peers, friends, and family [65]. Adolescents also report that significant barriers to help-seeking included embarrassment and perceived stigma, poor mental health literacy and problems identifying behaviors as harmful, along with the preference for self-reliance [65].

In conclusion, more work is needed to better understand factors associated with NSSI cessation and growth. We find NSSI cessation to be associated with a variety of factors across several psychosocial domains. Future research should examine these processes longitudinally in order to better inform prevention and intervention efforts.

## Abbreviations

NSSI: (non-suicidal self-injury), recovery, growth, NSSI cessation
